# On the Inside

**DOI:** 10.1093/plphys/kiab417

**Published:** 2021-10-29

**Authors:** Peter V Minorsky

**Affiliations:** School of Health and Natural Sciences, Mercy College, Dobbs Ferry, New York, USA

## Not all mobile mRNAs move the same

Long-distance mobile mRNAs play key roles in regulating various gene networks that control plant development and stress tolerance. Grafting experiments have confirmed that endogenous RNA molecules exist in phloem sap and mRNAs move in both the stock-to-scion and scion-to-stock directions. The long-distance transport of RNAs is facilitated by specific proteins that prevent RNAs from being degraded during transport. Lv et al. (pp. 1587–1604) now shed light on the species-specific differences in the movement of mobile mRNAs. They use grafts involving different apple (*Malus*) genotypes to demonstrate that apple (*Malus domestica*) oligopeptide transporter3 (*MdOPT3*) mRNA can be transported over a long distance, from the leaf to the root, to regulate iron uptake. In marked contrast, the mRNA of Arabidopsis (*Arabidopsis thaliana)* oligopeptide transporter 3 (*AtOPT3)*, the *MdOPT3* homolog from *A. thaliana*, does not move from shoot to root. When, however, *AtOPT3* was heterologously expressed in two woody species, *Malus* and *Populus*, it was able to move from the shoot to the root. In contrast, when the mobile homolog *MdOPT3* was heterologously expressed in two herbaceous species, *A. thaliana* and tomato (*Solanum lycopersicum*), its ability to move from the shoot to the root was lost. Previous research has shown that more than 10% of total phloem sap proteins are RNA-binding proteins (RBPs), and it is believed that the binding of phloem-residing RNAs to RBPs may play important roles in loading, unloading, and long-distance transport of RNA in the phloem sieve system. The authors demonstrate that the different transmissibility of *OPT3* in *A. thaliana* and *Malus* might be caused by divergence in RBPs between herbaceous and woody plants. This study provides insights into mechanisms underlying differences in mRNA mobility in plants.

## How the cucumber fruit gets its warts

The “wartiness” of cucumber (*Cucumis sativus*) fruits is an important quality trait that greatly affects fruit appearance and market value. Consumer preferences for warts or not depends on both culinary considerations and cultural traditions. A cucumber wart consists of a spine (a fruit trichome) in combination with an underlying tubercule, an arched structure derived from several layers of surface cells. The existence of spines is an epistatic prerequisite for tubercule formation. Although several regulators have been reported to mediate spine or tubercule formation, the direct link between spine and tubercule development remains unknown. Wang et al. (pp. 1619–1635) now show that the gene *HECATE2* (*CsHEC2*) is highly expressed in cucumber fruit peels including spines and tubercules. Knockout of *CsHEC2* by the CRISPR/Cas9 system resulted in reduced wart density and decreased cytokinin accumulation in the fruit peel. Conversely, overexpression of *CsHEC2* led to elevated wart density and cytokinin levels. Previous genetic studies showed that the warty phenotype is dominant over the non-warty fruit trait, and that fruit tubercule trait is controlled by a single dominant gene (*CsTu*), which encodes a transcription factor. CsTu apparently functions in cytokinin biosynthesis by indirectly promoting the expression of two cytokinin hydroxylase-like (*CHL*) genes, thereby stimulating cell division and ultimately causing the initiation of the fruit tubercule. Therefore, it is of interest that the present authors provide evidence that CsHEC2 directly binds to the promoter of the *cytokinin hydroxylase-like1* gene (*CsCHL1*), thereby activating its expression. Evidence is also presented that CsHEC2 physically interacts with GLABROUS3 (CsGL3, a key spine regulator). These data suggested that CsHEC2 promotes wart formation by acting as an important cofactor for CsGL3 and CsTu to stimulate cytokinin biosynthesis in cucumber.

## Auxin flow and endodermal development

The three specialized tissues of the roots of vascular plants, the epidermis, ground tissue (comprising the endodermis and cortex), and the vascular tissue, are derived from stem cell niches at the root apical meristem. The endodermis serves as a selective barrier that restricts the free diffusion of nutrients to the central vasculature. Seo et al. (pp. 1577–1586) now show that auxin is a key regulator determining the division of endodermal cells in Arabidopsis. Auxin induced the division of endodermal cells in wild-type plants, but not in the auxin signaling mutant *auxin resistant3-1* (*axr3-1*). The authors also report that the endodermis-specific activation of auxin responses by the directed expression of truncated *AUXIN RESPONSIVE FACTOR5* (*ΔARF5*), a more active form of the gene, in root endodermal cells also induced endodermal cell division. The auxin transport inhibitor 1-naphthylphthalamic acid (NPA) led to auxin accumulation in endodermal cells, which, in turn, induced endodermal cell division. In addition, knock-out of *P-GLYCOPROTEIN1* (*PGP1*) and *PGP19*, which mediate centripetal auxin flow, promoted the division of endodermal cells. The findings reported reveal a tight link between the endodermal auxin response and endodermal cell division, suggesting that auxin is a key regulator controlling the division of root endodermal cells, and that PGP1 and PGP19 are involved in regulating endodermal cell division

## Shoot herbivory impairs nematode root infection

Herbivore-induced defense responses are regulated by a network of interconnected signaling pathways in which plant hormones play a major regulatory role. Among them, the jasmonates, a family of oxylipins, have emerged as key signals in plant responses to chewing insect herbivores, such as beetles and caterpillars. Herbivore-induced defenses are typically expressed not only locally in the damaged tissue, but also systemically. The majority of studies on plant-mediated interactions between herbivores are constrained to aboveground tissues. A growing body of evidence, however, suggests that inducible systemic plant defenses can simultaneously influence the respective feeding rates of aboveground and belowground herbivores. However, the mechanisms driving these systemic effects and the long-distance signals involved remain poorly understood. Using tomato (*S. lycopersicum*) as a model plant, Martínez-Medina et al. (pp. 1762–1778) have explored the impact of leaf herbivory by the moth *Manduca sexta* on the performance of the root-knot nematode *Meloidogyne incognita*. They report that that leaf herbivory reduced *M. incognita* performance in the roots. By analyzing the root expression profile of a set of oxylipin-related marker genes and jasmonate root content, the authors demonstrate that leaf herbivory systemically activates the 13-Lipoxygenase (LOX) and 9-LOX branches of the oxylipin pathway in roots and counteracts the *M. incognita*-triggered repression of the 13-LOX branch. Further experiments revealed that having intact jasmonate perception in the shoot was essential for the inhibitory effects of moth shoot feeding on nematode root-feeding; intact jasmonate synthesis was not. These results highlight the impact of leaf herbivory on the ability of *M. incognita* to manipulate root defenses and point to an important role for jasmonate signaling in shoot-to-root signaling.

## Inhibited chloroplast N assimilation under high CO_2_

It is projected that global food production needs to be doubled by 2050 to meet demands. The improvement of photosynthetic efficiency is regarded as a major strategy for increasing crop yield potential. For C_3_ crops, one possible strategy for achieving this goal might be to inhibit photorespiration by increasing the CO_2_ concentration around Rubisco. Unfortunately, while increased atmospheric CO_2_ concentrations typically stimulate photosynthesis and crop yield in the major C_3_ cereal crops, they also decrease protein content. Insufficient leaf nitrogen (N) content can limit the development of sinks, such as grains, fruits, tubers, etc., which, in turn, causes feedback inhibition of photosynthetic gene expression. Insufficient N may also limit N investment into components of the photosynthetic apparatus, such as Rubisco and chlorophyll. To help make sense of this enormously complex problem, Zhao et al. (pp. 1812–1833) have developed a dynamic systems model of plant primary metabolism, which includes the Calvin–Benson cycle, the photorespiration pathway, starch synthesis, glycolysis–gluconeogenesis, the tricarboxylic acid cycle, and chloroplastic N assimilation. This model successfully captures responses of net photosynthetic CO_2_ uptake rate, respiration rate, and nitrogen assimilation rate to different irradiance and CO_2_ levels. The authors then applied this model to predict the inhibition of N assimilation under elevated CO_2_. Simulations suggested that enhancing the supply of α-ketoglutarate (2-OG) is a potential strategy to maintain high rates of N assimilation under elevated CO_2_. This model provides a basic framework for supporting the design and engineering of C_3_ plant primary metabolism for enhanced photosynthetic efficiency and N assimilation in the looming high-CO_2_ world.

## A small-molecule antagonist of jasmonic acid perception and auxin responses

In chemical genomics, large chemical libraries are screened to identify molecules reversibly affecting the activity of a protein, protein families, or a biochemical or signal transduction pathway. Hormone antagonists are invaluable tools for exploring the role of the hormone in specific tissues and developmental stages, and for identifying regulatory processes of the signaling pathway. The phytohormone jasmonoyl-L-isoleucine (JA-Ile) regulates many stress responses and developmental processes in plants. Using two complementary chemical screens, Chini et al. (pp. 1399–1413) searched for different and cheaper antagonists of JA-Ile perception. In this issue, they identify and characterize three commercially available JA-Ile antagonists. Of the three, the most intriguing was a molecule called J4 that exhibited JA-antagonist effects in planta, preventing several JA-mediated responses such as gene expression, growth inhibition, chlorophyll degradation, and anthocyanin accumulation in Arabidopsis, tomato (*S. lycopersicum*), and tobacco (*Nicotiana benthamiana*). In addition to antagonizing JA signaling, J4 also inhibited the closely related auxin pathway but no other hormonal pathways. Furthermore, the mode of action of this molecule is conserved in land plants, indicating its potential use in any plant species. This commercially available compound should prove to be a powerful tool for the pharmacological analysis and dissection of the JA and auxin signaling pathways.

